# Patterns of Polydrug Use in Patients Presenting at the Emergency Department with Acute Intoxication

**DOI:** 10.3390/toxics13050380

**Published:** 2025-05-07

**Authors:** Helena Manjón-Prado, Enrique Serrano Santos, Eduardo Osuna

**Affiliations:** 1 Department of Legal and Forensic Medicine, Biomedical Research Institute (IMIB), Regional Campus of International Excellence “Campus Mare Nostrum”, Faculty of Medicine, University of Murcia, 30120 Murcia, Spain; hmp.798@gmail.com; 2 Hospital Clínico Universitario Virgen de la Arrixaca, University of Murcia, Ctra. Madrid-Cartagena, s/n, 30120 Murcia, Spain; e.serranosantos@um.es

**Keywords:** epidemiology, emergency department, polydrug use, acute intoxication

## Abstract

Studies analyzing the prevalence of associated substance use are limited. Currently, the World Health Organization (WHO) defines polydrug use as the concurrent (simultaneous use) or sequential (use of one drug followed by another) abuse of more than one drug or type of drug, with dependence on at least one. Associated drug consumption can exacerbate the adverse effects and complicate the clinical management of patients. This study aimed to investigate the prevalence of polydrug use, excluding tobacco, in patients presenting with acute intoxication in the Emergency Department (ED) of the Clinical University Hospital Virgen de la Arrixaca (Murcia, Spain) in the year 2023. To this end, a retrospective analysis of 2562 patients was conducted, examining demographic variables, substance use patterns, reasons for presenting to the ED, and the substances consumed by each patient. The study reveals an average patient age of 41 ± 0.5 (SD = 11.96) composed of predominantly male patients (74.4%). A high prevalence of benzodiazepines and cocaine use, often in combination, was observed. The main reasons for attendance included symptoms such as palpitations, dyspnea, vomiting, diarrhea, behavioral disturbances, and self-harm. Only 25.5% of patients admitted to consuming all substances detected in their analyses. Polydrug use is frequent in our environment, which can lead to added complexity in diagnosis and treatment. Consumption patterns show a profile strongly related to the age of the subject. Among the youngest subjects, tetrahydrocannabinol (THC) and benzodiazepines predominate, whilst among older subjects, alcohol and benzodiazepines, and sometimes cocaine, predominate. This study highlights the need to design specific intervention and prevention strategies to address patterns of substance abuse, the importance of family and community support, and the need to tackle challenges in identifying and treating cases of polysubstance abuse. Moreover, cooperation between the healthcare system and law enforcement is also important to obtain up-to-date knowledge of new drugs and their consumption patterns in an emergency context.

## 1. Introduction

Drug abuse remains a major public health challenge globally, with significant clinical, forensic, and policy implications [1]. The illicit drug market continues to expand in both complexity and magnitude, with increasing availability of traditional substances and new psychoactive compounds.

A current problem is that preparations are produced containing one or more drugs other than the one the buyer expected, either mixed with the substance they intended to purchase or as a substitute for it. Therefore, the consumer may not know which substance or substances they are consuming. Furthermore, combined drug use, conscious or unconscious, may increase the risk of health problems due to pharmacological synergies or pharmacodynamic interactions and hinder the implementation of effective interventions in response to acute poisoning.

The European Drug Report of 2024 highlights the fact that the continuing health and security problems presented by established and newer illicit drugs, and increasingly, the interplay between them, create a challenging policy context for the shaping and implementation of effective responses [2].

The World Health Organization (WHO) defines multiple drug use as the concurrent (simultaneous use) or sequential (use of one drug followed by another) abuse of more than one drug or type of drug, with dependence on at least one [3]. A significant number of individuals treated for drug use present with combinations of substances, the most common being opioids, cannabis, and cocaine; cocaine, cannabis, and alcohol; and a stimulant, alcohol, and cannabis [4]. Furthermore, the European Monitoring Centre for Drugs and Drug Addiction (EMCDDA) [2] has identified three profiles among those seeking treatment: co-users of heroin and cocaine; co-users of cocaine, cannabis, and alcohol; and co-users of cannabis and alcohol. Alcohol is the most frequently present substance in polydrug use.

Despite the high rates of polydrug use, the literature on this matter remains limited. Furthermore, there are very few studies that analyze polydrug use according to diverse population features, particularly in clinical settings like emergency departments. Additionally, new substances and new patterns of use have emerged, posing significant clinical challenges [5,6,7,8,9] by exacerbating adverse effects and further complicating the clinical management of patients. A 2023 European Drug Emergency Network (Euro-DEN) Plus study demonstrated that polydrug use is a risk factor contributing to intensive care admission [10].

Cannabis is the most abused illegal substance. In some European countries, the use of specific types of medicinal cannabis and particular commercial goods incorporating cannabis by-products has been regulated. However, within the illegal drug trade, the prevalence of high-potency extracts and edibles is particularly alarming and has been associated with cases of acute toxicity in hospital emergency departments. Moreover, there is concern that some products sold on the illicit market as cannabis may be adulterated with potent synthetic cannabinoids [11].

The abuse of antidepressants is relatively rare in comparison to other prescription drugs, such as opioids or benzodiazepines. The misuse of benzodiazepines has been systematically reported over the years and is involved in approximately one-third of the unintentional drug overdoses. Its abuse usually takes place alongside the consumption of other substances such as alcohol, opioids, and stimulants [5,12,13,14,15,16]. Barbiturates were traditionally used as hypnotics, sedatives, antiepileptics, and anesthetics; however, their use is now limited to anesthesia and the treatment of epilepsy. The recognition of the risks of barbiturate dependence and toxicity, as well as the high incidence of poisoning, led to their replacement by benzodiazepines and Z-drugs [17,18].

Opioid use has been strongly growing in recent decades. A history of substance abuse or addiction is a risk factor for the development of opioid analgesic dependence in patients being treated for non-oncologic pain [19,20,21]. On top of this, frequent polydrug use has been reported in people with opioid use disorders, which, in turn, puts them at risk of suffering multiple substance use disorders [16]. The use of opioids alongside alcohol, amphetamines, or cannabis is very common [11,22,23].

In the case of cocaine use, prevalence has increased worldwide [24]. Its use is closely linked to nightlife, especially among the younger population [25]. Furthermore, one of the characteristics of its consumption is its high level of association with polydrug use, especially with alcohol, tobacco, cannabis, and, to a lesser extent, amphetamines, ecstasy, and heroin [9,26,27]. Therefore, high levels of cocaine commonly go hand in hand with high levels of these other drugs. However, the same is not true for methamphetamine, where data show that the more methamphetamine a user consumes, the less cocaine they consume, and vice versa [24]. In recent years, 3,4-methylenedioxymethamphetamine, commonly known as MDMA, “ecstasy”, “XTC” or “Molly”, has acquired a significant presence as a recreational drug in Europe and worldwide. Its use is predominant in the 15–34 age group [8]. The EMCDDA estimated that, in 2023, 4.3% of people aged 15–64 in the European Union had used MDMA at least once in their lifetime [2].

Polydrug use includes not only traditional illicit drugs, but also non-prescribed psychotropic medicines (N-PPMs) and new psychoactive substances (NPSs). These compounds possess potent psychoactive effects and are sometimes analogs to existing drugs and prescription products, and other times, they are newly synthesized chemical substances, created to simulate the psychoactive actions and effects of different products [28,29]. These new substances and preparations may exacerbate the emergence of new patterns of drug use since they are commonly presented in mixtures combining the new and the traditional drugs. However, due to the novelty of these substances, many hospitals are not yet able to analyze them, especially due to their large number, their chemical diversity and complexity, and the speed at which they appear on the market [29]. Therefore, our study focuses solely on the analysis of the consumption patterns of traditional drugs that are used in combination. The aim of this study was to identify which substances were most commonly co-used, excluding tobacco, among patients presenting with acute intoxication in the Emergency Department (ED), as well as the demographic characteristics of their consumers (gender and age) and aspects related to assistance.

## 2. Materials and Methods

### 2.1. Study and Sample Characteristics

In order to carry out the present research, a descriptive and observational retrospective study was conducted in patients who presented to the Emergency Department of the Clinical University Hospital Virgen de la Arrixaca (Murcia, Spain).

The study was conducted following the Declaration of Helsinki and approved by the Research Ethics Committee of the Virgen de la Arrixaca University Hospital on 9 May 2024 (approval number 1023/2024).

The initial study population consisted of all patients who presented to the ED during the year 2023, a sample size that was reduced according to the following established inclusion and exclusion criteria.

Inclusion criteria:

- Patients who tested positive for two or more substances and/or alcohol in the drug tests.

Exclusion criteria:

- Patients whose drug test results were negative or positive for a single substance/alcohol, since the focus of this study is polydrug consumption.
- Patients testing positive for two or more substances and/or alcohol, except tobacco, only due to previously prescribed drugs or medical treatment.

**Figure 1:**
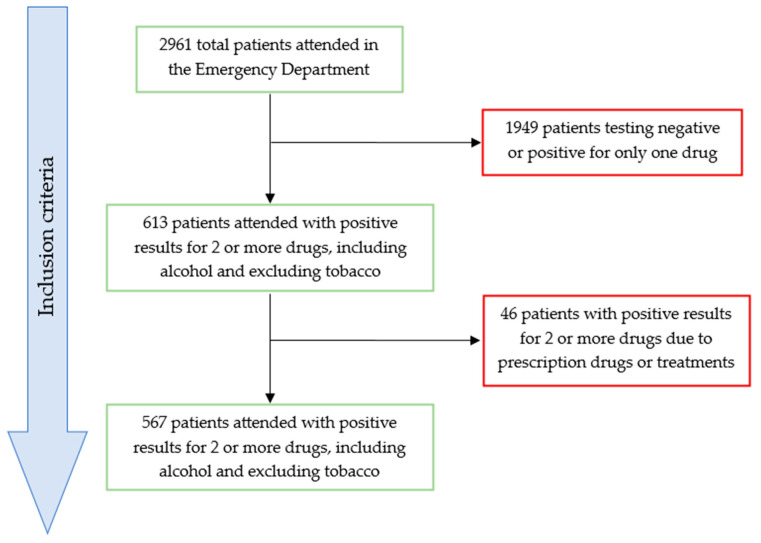
Figure showing the inclusion and exclusion criteria applied to the study population.

Thus, the final sample size consisted of 567 patients who met the inclusion criteria mentioned above (Figure 1). For these patients, their medical records were consulted to obtain additional information, as described in the following section.

The patients presented to the ED for acute intoxication. Blood and urine samples were drawn to determine the substance responsible for the intoxication. For these patients, their medical records were consulted to obtain additional information, as described in the following section.

Blood alcohol was quantified using a colorimetric enzymatic assay at 340 nm, using a Roche-COBAS diagnostic kit (Pleasanton, CA, USA). The ethanol present in the blood sample reacts with NAD^+^ and the enzyme alcohol dehydrogenase, producing acetaldehyde and reduced NADH, which is proportional to the amount of alcohol in the sample. Measuring Range (Serum/Plasma) 2.20–108 mmol/L (0.101–4.98 g/L, 10.1–498 mg/dL).

The drug analysis was carried out using the Multi-Drug One Step Multi-Line Screen Test Device in urine from the brand MONLAB (Barcelona, Spain). It is an immunoassay based on the principle of competitive binding. Drugs that may be present in the urine specimen compete against their respective drug conjugate for binding sites on their specific antibody. A drug, if present in the urine specimen below its cut-off concentration, will not saturate the binding sites of its specific antibody. The antibody will then react with the drug–protein conjugate, and a visible colored line will show up in the test line region of the specific drug strip.

The cut-off, sensitivity, and specificity values of the test are specified in Table 1.

### 2.2. Variables Analyzed

During the study, different variables were analyzed related to the substances analyzed, the patients’ gender and age, the time and date on which they presented to the ED, the reason for consultation, how the patient arrived and whether the patient came from a penitentiary center, as well as various aspects related to the substances consumed. For this purpose, the information of interest in the medical records was consulted using the SELENE application.

The categorization of the variables was carried out on the basis of the information collected and by dichotomizing some of the variables in order to facilitate the analysis and help in the reading and interpretation of the results. The established categories are age, gender, the presence or absence of alcohol, THC, opiates, morphine, methamphetamine, methadone, MDMA, phencyclidine, benzodiazepines, barbiturates, antidepressants, amphetamines and cocaine; whether the patient admitted the consumption of any of the substances in which they tested positive, what substances they consumed, what combinations of substances were consumed, at what time and date was the patient attended, the main reason for attendance, how the patient arrived to the Emergency Department and whether the patient was brought from a penitentiary center.

### 2.3. Statistical Treatment

The data were coded and processed using IBM SPSS Statistics 29.0.2.0. software for Windows. For the data analysis, descriptive statistics were applied by calculating frequencies and percentages for the dichotomized qualitative variables and mean and standard deviation for the quantitative variable age.

On the other hand, an inferential analysis was also carried out with contingency tables using Pearson’s Chi-square test or Fisher’s exact test in order to determine the degree of association between variables by running a corrected standardized residuals analysis of the interpretation of the association, considering a relationship between variables as statistically significant when the level of significance (*p*) was equal to or below 0.05. To establish the dependence between variables not due to chance, the criteria that all expected frequencies were greater than unity and that 20 were equal to or greater than 5 were applied. In the Results Section, a series of graphs resulting from the treatment of the data are presented.

## 3. Results

During the study period, 2961 people were treated in the ED for acute intoxication (Figure 1). The number of patients with positive results for two or more drugs, excluding tobacco, was 567, of whom the majority, 74.4%, were male. The mean age was 41 ± 0.5 years (SD = 11.96), with a range going from 15 to 77 years (Table 2). In our study, the group of minors consisted of only nine individuals, and the group of patients over 70 years old consisted of only six individuals, so we cannot draw any conclusive results regarding these groups due to the small number of cases.

In relation to the main reasons for attendance, the most common were symptoms such as palpitations, dyspnea, vomiting, nausea, diarrhea, sweating, etc. (20.1%), followed by behavioral disturbance (19.4%), self-harm (15.7%), and altered consciousness (14.8%).

Most patients tested positive for two substances (58.7%). The number of patients testing positive decreased as the number of associated substances increased, with only one person testing positive for seven substances, these being THC, opiates, morphine, methamphetamine, MDMA, amphetamines, and cocaine. In the patients who attended by polydrug users, the most common substances were benzodiazepines (74.2%), alcohol (61.3%), and cocaine (58.4%), as shown in Table 3.

In our study, we identified 95 different combinations of substances. The most frequent are benzodiazepines with cocaine (18.7%), alcohol with benzodiazepines (16.4%), THC with benzodiazepines (15.6%), THC with benzodiazepines and cocaine (14.8%), and, finally, THC with cocaine (10.6%) (Table 4).

In addition, we also found that the most frequent combination of drugs in women was THC with benzodiazepines (13.1%), whilst for men it was benzodiazepines with cocaine (14.0%).

Table 5 shows the pairs of substances that exhibit a statistically significant relationship (p≤0.05).

With respect to the demographic variables, patients who use benzodiazepines with cocaine are mostly male (81.9%), between 40 and 49 years of age (37.5%), and who arrive at the ED for reasons related to self-harm or behavioral disturbance (19.4% in both cases). The patients who combine alcohol with benzodiazepines are frequently male (69.8%) with ages between 50 and 59 years (36.5%), who arrive at the ED due to reasons related to self-harm (27.0%). Lastly, in the case of those who mix THC and benzodiazepines, they are usually between 18 and 29 years old (35.0%), commonly men (68.3%), with symptoms related to other diseases not related to drugs (31.7%).

When asked about the substances consumed, 25.2% of patients who tested positive for polydrug use admitted the consumption of all substances detected, while 27.3% admitted the intake of some substances but not all of them. The remaining 47.4% denied or did not mention any of the substances used.

The use of THC with benzodiazepines is more common among younger age groups. In the 18- to 29-year-old group, the prevalence is 22.3%, followed by the combination of THC and cocaine (16.0%). For patients aged 30–39 years, the most frequently used combination of substances was THC, benzodiazepines, and cocaine (22.3%), followed by benzodiazepines and cocaine (13.8%). In the 40–49 age group, the most common combination is benzodiazepines with cocaine, accounting for 14.9% of the cases, followed by alcohol and benzodiazepines (10.5%). Lastly, for those aged 50 and above, the most common combination of substances is alcohol and benzodiazepines.

The Chi-square statistical test for each substance with age shows there is a statistically significant relationship (p≤0.05) between age and the use of THC, morphine, benzodiazepine, and cocaine, as is shown in Table 6.

## 4. Discussion

In this research, the findings provide a comprehensive overview of polydrug use and their association with age, gender, reason for consultation, and other relevant variables in patients who attended an ED. Our sample showed a predominance of male patients (74.4%) with a mean age of 41 ± 0.5 years (SD = 11.96). These results are in line with other studies indicating higher substance use among males than females [9,30,31,32].

The predominance of men among patients may reflect a higher prevalence of substance use in general in this population, possibly influenced by socio-cultural factors that normalize drug use among males [33]. According to the 2023 report of the Spanish Observatory on Drugs and Addictions (OEDA), prevalence rates are higher in the 15–34 age group, except for drugs with addictive potential, such as opioid analgesics and hypnosedatives, where the highest prevalence rates are observed in the older age groups [34]. A study carried out in Mexico obtained similar results, with the 18–34 age group being the population with the highest consumption rates [35]. Due to the large number of patients in this study who ingested benzodiazepines, the results obtained may have been biased towards a slightly higher mean age.

In turn, the main reasons for attendance were symptoms such as palpitations, dyspnea, vomiting, diarrhea, behavioral disturbance, and self-harm. Addiction to toxic substances is known as a relevant factor triggering antisocial and/or violent behavior [36,37]. For example, several studies highlight the existence of a positive correlation between the consumption of cocaine and the development of violent behaviors, as well as the severity of these [9,31,38].

The high prevalence of benzodiazepines and cocaine, as well as their frequent combination, suggests the need for specific intervention strategies to address these consumption patterns. In Spain, there is an increasing trend in the use of hypnosedatives; on the other hand, the prevalence of cocaine use is also high [39], making it the second most common substance among patients entering detoxification treatment. These data suggest a high prevalence of both substances independently, which could indicate a possible coexistence of consumption in some groups of users. The association of these two substances could suggest a pattern of use oriented towards self-medication to treat symptoms of anxiety and sleep disorders [40] or the search for synergistic effects, indicating a pattern of concurrent use aimed at balancing the effects of excitation and relaxation. Also, it is important to note that a positive result indicates the presence of the tested compound or its metabolites but provides no information on how the drug was administered, the concentration of the drug in the urine, or the last exposure. Furthermore, benzodiazepines can produce positive results in these tests long after use. This mix of positivity might be due to recent concomitant use or multiple exposures to both substances, or it could be a consequence of long-term benzodiazepine use, which can be detected in the medium and long term during the clinical care of patients with acute cocaine use.

On top of this, there is a high prevalence of benzodiazepine prescription to older people for the treatment of insomnia and anxiety [5,41] and, despite the known dangers, this prevalence increases with the age of the patients [42]. To further complicate things, the prescription of multiple drugs to the elderly is frequent and can cause drug–drug interactions that can lead to toxicity [43].

In addition, the frequent combination of THC with other substances could be related to its widespread recreational use and easy availability, and the potential to enhance the effects of other drugs. Moreover, THC is one of the most widely consumed drugs among young people. Due to this, prevention and treatment programs should focus on the most common combinations of substances and educate about the risks of their associated and concurrent use, especially among the younger population who may be more vulnerable [44].

In terms of substances that patients admitted to using, 25.2% of the patients who tested positive for polydrug use admitted to using all the substances detected, while 27.3% admitted to using some but not all of the substances detected. Among all the substances mentioned, the majority were alcohol and benzodiazepines. This again highlights the problem of the abuse of benzodiazepines prescribed for self-medication. In the case of alcohol consumption, a study conducted in Japan by Osaki et al. [45] showed that it is more common in men than in women and that, in addition, the starting age of alcohol consumption is increasingly younger. Similarly, a review article on the current situation in Spain [46] points out that Spain is one of the countries with the highest prevalence of alcohol-related problems compared to the rest of Europe. According to the data, 93.0% of the population between the ages of 15–64 has consumed alcoholic beverages sometime in their lifetime, and the prevalence of daily alcohol consumption is 8.8% in the general population. The study also highlights that, independently of gender, acute alcohol poisoning is more prevalent in the 15–34 age group. It is therefore important to develop and implement educational programs aimed at different age groups, focusing on the risks of individual drug use, the risks of mixing certain substances, and the importance of seeking professional help.

Finally, the wide variety of substance combinations highlights the need to improve diagnostic and treatment capabilities in the Emergency Department in order to ameliorate therapeutic guidelines for patients presenting with intoxication for this reason. Therefore, continuous training of healthcare workers is essential to identify and effectively manage cases of polydrug use. Moreover, it also seems necessary to encourage collaboration between health services, police force, and other agencies to provide a comprehensive and coordinated response to substance misuse cases, so as to have the most up-to-date knowledge possibly of the new polydrug combinations being used nowadays [47].

## 5. Strengths and Limitations

The main strength of this study lies in the fact that it was conducted among the entire population treated for acute intoxication in the ED of a regional referral hospital with 920 beds. Therefore, the results obtained can be extrapolated, with the limitations outlined below, to the reality of the phenomenon.

However, the findings must be interpreted within the context of the study’s limitations. We have collected information from all patients who have come to the ED, but it is a retrospective study, and it is important to highlight the difficulty, at times, of extracting the information on which this article is based, as it is collected from medical records. The exact drug composition, exposure dose, mode of use, and pattern of use were not collected in some cases. We have studied the polydrug phenomenon, but we have only analyzed the results obtained from traditional drugs. There is a large number and variety of substances that can be found as NPS, which we were not able to include, as they are not analyzed in our hospital. The novelty of these drugs poses difficulties in their determination.

Additionally, in some cases, reliance on self-reported data may affect the accuracy of our findings on use patterns and associations between different substances. Missing or misreporting, whether intentional or due to a lack of knowledge about all substances used, could lead to biased estimates of prevalence and associations between substances used. This limitation could obscure certain associations or exaggerate others, which could somewhat reduce the reliability of conclusions about the relationship between co-intoxications.

## 6. Conclusions

The association of substances is frequent in our environment, which can lead to added complexity in the approach to this phenomenon, as well as in its treatment. In our study, a high prevalence of association between substances was observed, with a high prevalence especially in the use of benzodiazepines, cocaine, and THC, commonly in combination. In patterns of use in which several substances are associated, the most frequent combinations are benzodiazepines and cocaine, mostly in men aged 40–49 years and within a context of behavioral disturbance or self-harm; alcohol and benzodiazepines, in men aged 50–59 years for reasons of self-harm; and THC and benzodiazepines, mostly in men aged 18–29 years. Consumption patterns also show a profile strongly related to the age of the subject. Among the youngest subjects, THC and benzodiazepines predominate, whilst among older subjects, alcohol and benzodiazepines, and sometimes cocaine, predominate.

This study underlines the complexity of polydrug use and the need for a multidisciplinary approach to addressing this public health problem. Intervention strategies should be comprehensive, including prevention, social support, and education, as well as the development of protocols for data collection and substance testing in a clinical setting, enabling a specialized approach to reduce the negative impact of polydrug use on the health of individuals and society.

## 7. Future Research

Future research should aim to improve detection methods by adapting toxicological screening procedures to keep pace with the rapid emergence of new psychoactive substances on the drug market. The dynamic nature of substance use patterns, driven in part by the appearance of novel compounds and mixtures, requires analytical tools that are both sensitive and up to date, in order to ensure accurate clinical assessment and public health monitoring.

Additionally, further studies are needed to better understand the difference between individuals who engage in single-substance use and those who engage in polydrug use. Exploring these differences in terms of clinical presentation, health outcomes, and treatment needs could provide valuable insights for tailored interventions.

Finally, replicating this study with a specific focus on adolescents and minors is essential. Even though our current sample included patients under 18, the number was too small to draw any statistically significant conclusions. Given the increasing prevalence of substance use in younger populations, dedicated research on this demographic is warranted to inform age-appropriate prevention and intervention strategies.

## Figures and Tables

**Table 1:** Table showing the cut-off, sensitivity, and specificity values of the drug test.

Drug	Cut-Off (ng/mL)	Sensitivity (%)	Specificity (%)
Amphetamine (AMP)	1000	94	99
Barbiturates (BARs)	300	92	99
Benzodiazepines (BZDs)	300	98	99
Cocaine (COC)	300	95	>99
Marijuana (THC)	50	95	96
Methadone (MTD)	300	93	>99
Methamphetamine (MET)	1000	90	>99
MDMA (Ecstasy)	500	99	99
Morphine (MOP 300)	300	98	98
Opiates (OPI 2000)	2000	98	97
Phencyclidine (PCP)	25	99	98
Tricyclic Antidepressants (TCAs)	1000	96	94

**Table 2:** Table showing the number of patients per age group.

Age Group	Number of Patients
Minors	9
18–29 years	94
30–39 years	130
40–49 years	181
50–59 years	118
60–69 years	27
≥70 years	6

**Table 3:** Table showing the percentage of patients who tested positive for each individual drug.

Drug	Percentage of Patients Who Tested Positive (%)
Alcohol	61.3
THC	47.4
Opiates	13.9
Morphine	15.5
Methamphetamine	3.6
Methadone	8.4
MDMA	4.6
Phencyclidine	0.6
Benzodiazepines	74.2
Barbiturates	0.5
Tricyclic Antidepressants	6.4
Amphetamines	6.2
Cocaine	58.4

**Table 4:** Table showing the 10 most frequent substance combinations.

Associated Drugs	Percentage (%)
BZDs + Cocaine	18.7
Alcohol + BZDs	16.4
THC + BZDs	15.6
THC + BZDs + Cocaine	14.8
THC + Cocaine	10.6
Alcohol + Cocaine	7.5
Alcohol + BZDs + Cocaine	6.2
THC + Opiates + Morphine + BZDs + Cocaine	3.6
Alcohol + THC	3.4
BZDs + Tricyclic Antidepressants	3.1

BZDs = Benzodiazepines; THC = Tetrahydrocannabinol.

**Table 5:** Table showing the associated substances whose relationship is statistically significant and their corresponding Chi-square (χ^2^) and critical level of probability (*p*).

Associated Substances		Χ^2^	*p*
THC	Benzodiazepines	6.428	0.011
THC	MDMA	3.894	0.048
THC	Methamphetamine	4.324	0.038
Opiates	Methadone	7.676	0.006
Opiates	Morphine	364.415	<0.001
Morphine	Methadone	18.800	<0.001
Methamphetamine	Amphetamines	113.714	<0.001
Methamphetamine	Benzodiazepines	16.412	<0.001
Methadone	Benzodiazepines	4.862	0.027
MDMA	Amphetamines	120.209	<0.001
MDMA	Benzodiazepines	19.495	<0.001
Phencyclidine	Cocaine	4.131	0.042
Benzodiazepines	Cocaine	16.458	<0.001
Benzodiazepines	Amphetamines	5.799	0.016
Benzodiazepines	Antidepressants	4.967	0.026
Antidepressants	Cocaine	16.054	<0.001

**Table 6:** Chi-square (χ^2^) values for drugs and age.

	Age	
Drug	χ^2^	*p*
THC	50.520	<0.001
Opiates	10.962	0.090
Morphine	12.915	0.044
Benzodiazepines	31.316	<0.001
Cocaine	41.915	<0.001

Only the cases where the minimum expected value is greater than 1 are shown.

## Data Availability

The data presented in this study are available upon request from the corresponding author due to ethical and privacy restrictions. Anonymized datasets may be provided upon request and approval by the corresponding ethic committee.

## Abbreviations

The following abbreviations are used in this manuscript:

ED	Emergency Department
EMCDDA	European Monitoring Centre for Drugs and Drug Addiction
Euro-DEN	European Drug Emergency Network
INCB	International Narcotics Control Board
MDMA	3,4-methylenedioxymethamphetamine
NPS	New Psychoactive Substances
N-PPM	Non-prescribed psychotropic medicines
OEDA	Spanish Observatory on Drugs and Addictions
SD	Standard Deviation
THC	Tetrahydrocannabinol
WHO	World Health Organization
